# Analysis of RFI Identification and Mitigation in CAROLS Radiometer Data Using a Hardware Spectrum Analyser

**DOI:** 10.3390/s110303037

**Published:** 2011-03-07

**Authors:** Pascal Fanise, Mickaël Pardé, Mehrez Zribi, Monique Dechambre, Christophe Caudoux

**Affiliations:** 1 LATMOS, Route de Troux, 78280 Guyancourt, France; 2 CESBIO (UPS/CNRS/IRD/CNES), 18 Avenue Edouard Belin, bpi 2801, 31401 Toulouse Cedex 9, France

**Keywords:** RFI, CAROLS, L-band, radiometry

## Abstract

A method to identify and mitigate radio frequency interference (RFI) in microwave radiometry based on the use of a spectrum analyzer has been developed. This method has been tested with CAROLS L-band airborne radiometer data that are strongly corrupted by RFI. RFI is a major limiting factor in passive microwave remote sensing interpretation. Although the 1.400–1.427 GHz bandwidth is protected, RFI sources close to these frequencies are still capable of corrupting radiometric measurements. In order to reduce the detrimental effects of RFI on brightness temperature measurements, a new spectrum analyzer has been added to the CAROLS radiometer system. A post processing algorithm is proposed, based on selective filters within the useful bandwidth divided into sub-bands. Two discriminant analyses based on the computation of kurtosis and Euclidian distances have been compared evaluated and validated in order to accurately separate the RF interference from natural signals.

## 1. Introduction

In recent years, several studies have revealed the highly detrimental influence of radio frequency interference (RFI) on microwave observations of the Earth, especially in the case of spatial radiometry [1]. This kind of perturbation corrupts the recorded signals, thus deteriorating the data quality to variable degrees which, in some cases, render it unusable. The presence of strong RFI is particularly noticeable over continental surfaces, but has also been observed by the recently launched SMOS satellite over the ocean [2].

At low frequencies (L-band), the soil moisture (SM) fraction has a negative-signed dependence on the brightness temperature (Tb) of 1 K/%, with its exact value depending on soil type, vegetation cover, surface roughness and other factors. The SMOS radiometer has been designed to measure the surface soil moisture, with a specified radiometric error of less than 2 K, depending on the nature of the target and its position in the instrument field of view. The SM retrieval error is predicted to be lower than 4%, as required by the hydrology community [2]. Small levels of RFI, producing only a few Kelvin in terms of equivalent radiometric signal, would thus cause the soil to appear dryer, which in turn would be interpreted as lower recent rainfall, or higher recent evaporation. The large spatial scale of satellite measurements could be a second factor contributing to an increase in the presence of RFI.

The future SMAP satellite [3] will benefit from current RFI studies: this mission is designed to analyse the full protected bandwidth (which cannot be done by SMOS), in order to mitigate RFI effects on brightness temperature (Tb) measurements, by identifying and eliminating the sub-bands which are affected by RFI.

RFI detection and mitigation have already been studied and reported in several papers, most of them are related to radio astronomy [4] or C-band [5] and L-band [6–8] Earth surface remote sensing; among those studies, several are based on the analysis of real airborne data [9–11]. Recently, different methods were developed and evaluated, based on the identification of RFI in radiometric measurements. The analysis of kurtosis thresholds applied to Tb measurements [12], or to the first and second U, V Stokes parameters [9,10], has also been evaluated for contaminated data detection in the time domain.

An alternative approach, in the frequency domain, is based on the identification of RFI in the radiometric spectrum [13–15], followed, if possible, by a spectral blanking, *i.e.*, the elimination of the corresponding contaminated data from the spectrum. It is hoped that this approach, which is an attempt to preserve only the cleaned data, will drastically reduce the amount of radiometric data lost in spatial missions such as SMAP.

Various studies based on the spectral analysis of RFI signals have been made in the past. In [6] the authors demonstrated the ability of an ADD (Agile Digital Detector) to detect and remove RFI from microwave measurements. The ADD performance was experimentally verified under controlled laboratory conditions, and in the field near to commercial air traffic control radar. In [7], the authors tested a double RFI detector based on a kurtosis parameter computation, under equivalent conditions. These tests have been conducted with data acquired from ground-based radiometers or from laboratory measurements. In addition, recent airborne radiometer campaigns were carried out with the aim of RFI surveys in the USA [16].

In France, in the context of validation campaigns for the SMOS mission, the CAROLS L-band radiometer [17] was installed onboard the French ATR-42 research aircraft and operated during the 2007 to 2010 “CAROLS campaigns”. These first campaigns were dedicated to the qualification and certification of the instrument and to the validation of the data quality, and, at the end, to the acquisition of different types of brightness measurements over oceanic and land surfaces (in the south west of France). During all the different flights, mainly over the land, we observed the presence of high level RFI perturbations, continuous as well as pulsed. These perturbations were not really expected because of the supposed protected observation bandwidth. We analyzed the corrupted signals and attempted to mitigate them [10]. In addition to our analysis, we decided to build and add the CAROLS airborne radiometer, and operated this new system during the last flight of the last campaign. In this paper, we present a study that we have conducted in order the analyze the RFI present in the L-band spectrum (1,400–1,427 MHz) using this small but helpful database. The aim of this study was the validation of the new analyzer rather than the validation of the algorithms used to interpret the data, and more particularly the data acquired close to high RFI areas

Although instruments obtaining similar spectrally-resolved data have been deployed in previous more extensive airborne campaigns, the results to be shown, though limited, are of interest because they are measured in geographic areas in that have note been studied previously with a radiometer having spectral resolution.

Our paper is organized as follows: Section 2 presents the radiometer and spectrum analyzer system, Section 3 presents our analysis of the radiometric data, and more particularly the data acquired close to high RFI areas, and Section 4 presents two different RFI mitigation algorithms based on subband filtering. All of these were tested with the CAROLS 2009 database, the results of which are presented in Section 5. Finally, our results are discussed in Section 6.

## 2. The CAROLS Radiometer and the Added Spectrum Analyzer System

### 2.1. CAROLS Radiometer

The receiver was designed and built as a copy of the EMIRADII [18] radiometer constructed by the DTU (Danish Technical University) team, and was adapted to the French ATR42 research aircraft. It is a fully polarimetric correlation radiometer with direct sampling (using the subsampling concept described in [18]), enabling the measurements of the four Stokes parameters describing the observed electric fields. The detailed characteristics of CAROLS were presented in [17], and only its main characteristics are summarized here. The radiometer is calibrated using internal loads and a pulsed noise diode, which adds approximately 150 K to its input. This setup ensures the system ability to carry out regular internal calibrations, and allows the radiometer to calibrate any phase difference between the two input channels preceding the digital correlator.

The stability, accuracy and linearity of the radiometer were fully analyzed in the laboratory (see Zribi *et al.*, [17]) using a cryogenic load, and lead to the specifications described in Table 1.

### 2.2. Spectrum Analyzer System

**Figure 1.**
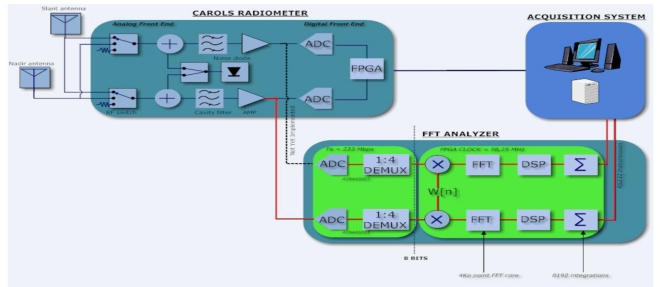
Block diagram of the CAROLS radiometer and (FFT) spectrum analyzer. The red connection was not fully operational during the 2009 CAROLS campaign.

The architecture of the spectrum analyzer (SA) developed for the CAROLS campaigns is presented in Figure 1. The first element is a high resolution analog to digital converter (ADC), followed by a field programmable gate array (FPGA) whose role is to perform digital signal processing.

The SA uses the principle of direct RF sampling. The analog signal is bandwidth limited and it can be sampled and digitized immediately after the front end amplification and filtering stages. This technique is used to replace the use of mixer and oscillator local by a very large wideband analog to digital converter (ADC). Then, the band of interest (1.4–1.427 GHz) can be translated down to 2–29 MHz by using a frequency clock to 233 MHz. The converter clock has been carefully chosen with a phase noise below 150 dBc/Hz (@ 10 MHz) to conform to the ADC specification. The de-multiplexed output is allowed to keep only one sample out of four after digitization. The RF signal can be perfectly reconstructed, without loss of information, if the sampling frequency is greater than twice the bandwidth of the signal being sampled, according to Nyquist principle.

Following this first step, the demux output is multiplied by a symmetrical window function (*i.e*., a Hamming function) to prevent truncation artifacts. The digital signal processing consists in the computation of a Fast Fourier transform (FFT), followed by the power spectrum estimation and accumulation. The sampling rate into the FPGA is set to 58.25 MHz. A 4K FFT process takes place into the FPGA, each FFT is then performed in 70.32 μs. The frequency resolution is 14.22 KHz. We have chosen 1,024 integration, the FPGA was providing a “mean FFT” every 72 ms.

Connected to the H channel of the CAROLS radiometer slant antenna during the present campaign, the SA allows one to record the modulus of integrated spectra, according to the specifications described in Table 2 below.

## 3. Presentation of the Radiometric Data and Spectra

### 3.1. CAROLS Flight

**Figure 2.**
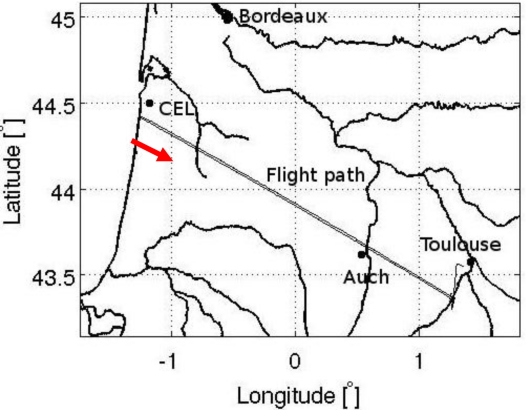
CAROLS flight path on 29 May 2009. The portion of the flight indicated by the red arrow was dedicated to the use of a spectrum analyzer, in combination with the CAROLS radiometer.

A special flight of the ATR42 research aircraft, operated by SAFIRE, was carried out on 29 May 2009. Figure 2 illustrates the flight path over southwest France. The aircraft flew at a constant altitude of 3,000 m above the ground between Toulouse and the Atlantic Ocean which are about 235 km apart. A large amount of measurements made along the transect was disturbed by interference in both H and V polarizations [10]. Pulsed RFI sources appeared clearly during most of the flight, as well as two point-like sources producing continuous-wave (CW) RFI, which strongly disturbed the radiometric measurements. The latter sources were situated close to the city of Auch, and to the military “Centre d’Essai des Landes” (CEL, *Moorlands Test Centre*) area in the vicinity of the western extremity of the flight path (south of Bordeaux).

During this flight, radiometric spectra were acquired during both legs of the journey (from Toulouse to the Atlantic and from the Atlantic back to Toulouse), using two sampling frequencies. For the purposes of this study, we chose to analyze the data corresponding to the second part of the flight only, in order to ensure homogeneity of the analysis. Moreover, this leg of the flight was more strongly disturbed by RFI, as the slant antenna was facing towards the south where the sources were situated.

### 3.2. Data from the Spectrum Analyzer

**Figure 3.**
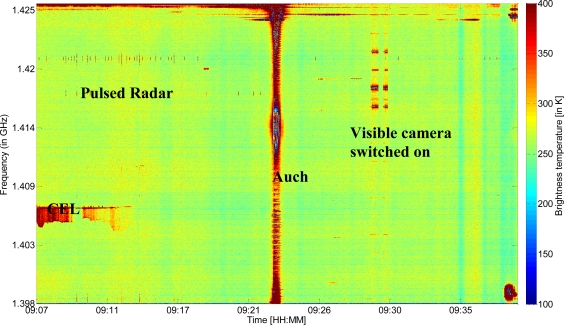
Time-Frequency representation of calibrated Tb values, during the flight from the Atlantic Ocean to Toulouse city (France).

Figure 3 represents the data collected during the spectral analysis flight. To plot this figure, each frequency channel was first calibrated: we used samples acquired regularly looking to an internal load source (∼310 K), with and without an internal noise source (adding 140 K), which allow us to have two points for calibration line definition and then Tb estimation. An external cryogenic load was used for the noise source evaluation and for cable loses estimation. The spectral variations were plotted as a function of time from the ocean to Toulouse (from the left to the right of the figure).

During land survey several peaks corresponding to the presence of three distinct types of RFI can be identified: pulsed RFI sources in the frequency range 1.418–1.419 GHz, emitted by military and civil radars, CW RFI in a narrow band (CEL), and CW RFI occupying the full bandwidth (Auch). Because the latter form of RFI is distributed over the full bandwidth of interest, it was not possible to restore the correct values for Tb. Between 9:07 and 9:23, disturbance is present near 1.426 GHz, probably due to the antennas situated in the vicinity of Auch. These antennas were identified and are known to have a central emission frequencies between 1,427.1 and 1,427.9 MHz, with H or V polarization.

Additional disturbances can be seen between 9:28 and 9:30, corresponding to the switching on and off of a visible camera, thus revealing the strong sensitivity of radiometric measurements to other onboard instruments. Moreover, vertical and horizontal strips are observed on Figure 3 independently to the RFI. The vertical ones, which are not regularly spaced in time, are probably due to intrinsic variations of natural Tb. The horizontal ones are due to the calibration versus frequencies which varies slightly from one frequency to another.

Some other features can be seen on this figure where no obvious RFI is present. Some of these vertical features are due to natural variations; the horizontal one could be due to the independent calibration for each channel.

**Figure 4.**
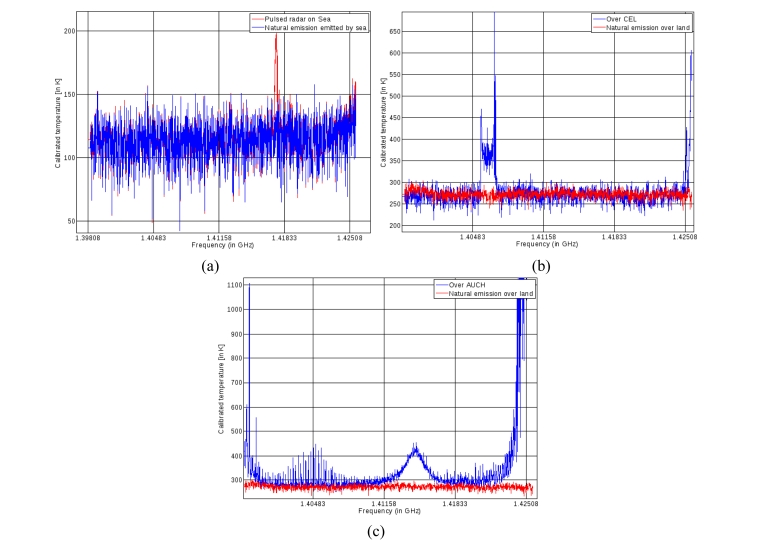
Spectral samples corresponding to **(a)** Pulsed RFI, **(b)** full band CW RFI and **(c)** narrow band CEL RFI. In each figure, the red curve corresponds to the unpolluted (natural) radiometric spectrum.

In Figure 4(a), a pulsed RFI source is represented during the ocean survey—just before the beginning of the transect shown in Figure 3 (we selected these spectrum over sea because it is easier to discriminate small pulsed RFI from natural emission). A peak of 50 K, centered on 1.418 GHz, is clearly visible. This pulse occurred several times during the flight, and its intermittent spectrum can be seen in the left half of Figure 3 at the same frequency.

Figure 4(b,c) illustrates calibrated temperature values as a function of frequency, for the two areas corresponding to Auch and the CEL, respectively. Concerning the perturbations detected in the Auch area, strong RFI can be seen over the entire bandwidth, with a very strong peak at 1.415 GHz. We observe RFI in this location as a consequence of the presence of two transmitters with central frequencies close to 1.428 GHz. These types of transmitter, emitting frequencies close to those of the protected L-band, are present throughout France. A map of their locations is provided in [10]. In the case of the CEL perturbations, the RFI is limited to a small band centered around 1,406 MHz, with an approximately 3 MHz bandwidth. The brightness temperatures over the remaining parts of the spectrum appear to be close to 260 K, which should correspond to natural emission.

## 4. RFI Mitigation Algorithm Using Subband Filtering

In this section we present two algorithms, designed to eliminate those parts of the spectrum corrupted by RFI. We applied in order to recover Tb values free from the influence of RFI. In order to identify the presence of RFI. An easy approach consists in dividing the spectrum into different sub-bands, and then applying different criteria to each subband, so as to classify the samples according to whether or not they are affected by RFI. The application of these algorithms requires the definition of thresholds over which samples are supposed to be corrupted. Each threshold was chosen empirically after different tests applied on the whole data set.

### 4.1. Algorithms Based on the ‘Gaussianity’ Hypothesis: Generalized Spectral Kurtosis

Kurtosis [19] is defined as the normalized form of the fourth central moment *μ*_4_ of a distribution, expressed by the ratio: $\beta_{4}=\frac{\mu_{4}}{\mu_{2}^{2}}$ where *μ*_2_ is the second central moment and *μ*_4_ is the fourth central moment of a statistical distribution. *β*_4_ is equal to 3 for a Gaussian distribution (natural emission), and the deviation of this ratio from its constant value of 3 is an indicator of the presence of non Gaussian noise (RFI). For example, the kurtosis of pulsed RFI with a duty cycle lower than 50% is greater than 3, and this property can be used to flag an RFI-contaminated subband, using a so-called generalized spectral kurtosis (SK) parameter [20,21]. This criterion can be applied to each of the sub-bands, for every radiometric sample. In the present study, we chose to eliminate data whose spectral-kurtosis was greater or lower than four times the standard deviation (STD).

A drawback of using the kurtosis parameter is that it has a blind spot for sinusoidal and chirp interference signals with a 50% duty cycle. Nevertheless, as a kurtosis algorithm was found by [22] to be the most suitable for normality analysis, we decided to test this method in our study, despite the limitation resulting from a low number of samples. For these algorithms we divided the spectrum in only eight sub-bands to evaluate the ‘gaussianity’ of each sample.

### 4.2. Algorithm Based on the Euclidian Distance

The Euclidian distance [23] is a useful technique for determining the similarity of a sample to a set of values measured from a collection of known samples (training basis). In our application, the known samples are those considered to be uncorrupted by RFI (clean samples). The greater the Euclidian distance between the clean and the evaluated samples, the greater the likelihood of the evaluated sample is interpreted as interference noise. The Euclidian distance is defined as:

(2) $$D_{M}\;\left(x\right)=\sqrt{{\left(x-\mu \right)}^{T}\left(x-\mu \right)}$$

where μ is the mean signal strength of a clean sample, and x is the evaluated sample. Once the clean samples (μ) have been empirically determined, we compute the D_M_ values for each sub-band of all the spectra, the average (mD_M_) and standard deviation (sD_M_) of all D_M_, and then apply a mask to the data for which the values of D_M_ exceed mD_M_ + 2 sD_M_ .

One drawback of this method (that was already proposed and tested in [14]) is the need to define one (or many if ocean survey data are present in the database) RFI-clean database, before evaluating the extent to which the remaining data is corrupted. This requirement makes the Euclidian method difficult to apply during operational procedures. It was nevertheless tested during our campaign, as it has the potential of providing a good comparative reference, which could be used to evaluate the efficiency of sub-band division.

## 5. Results

The comparison of different RFI mitigation algorithms, using real data, is not a straightforward task. In the absence of an ‘*a priori*’ knowledge of the data corruption level and the brightness temperature of the natural scene, we can only use indirect methods to estimate the efficiency of these algorithms. Nevertheless, one possible approach is to visually characterize this efficiency, since strong RFI can easily be identified. Additional information is provided by the quantity of data remaining, after elimination of the samples found to be RFI; a further clue is given by the mean of the final Tb value, which should be as low as possible.

### 5.1. Kurtosis Mask

**Figure 5.**
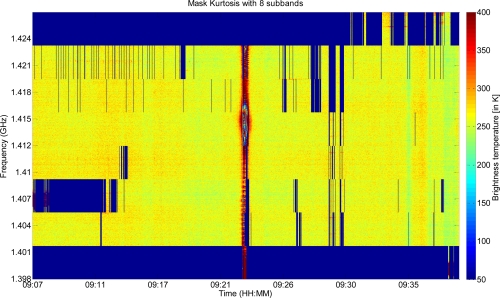
Spectral filtering using the kurtosis filter, and with the spectrum divided into 8 sub-bands.

In the case of the kurtosis algorithm, we computed the kurtosis for each subband. The sub-bands exceeding the applied threshold were not included in the brightness temperature computation. The kurtosis algorithm needs a large amount of data, to be efficient. In our study, with 8 sub-bands, we were limited to 256 values per subband, and it was not possible to increase the number of sub-bands without damage. This is a significant limitation of this criterion. The results obtained with this method are shown in Figure 5.

The RFI present at the higher frequencies is almost always detected, as is the pulsed RFI, but it can be seen that the narrow band RFI transmitted by the CEL is not completely detected. Moreover, some parts of the spectrum collected near Auch (in the middle of the figure) are also not discriminated. Nevertheless it appears that very few false alarms are generated when using this filter, with the spectrum divided into eight sub-bands.

### 5.2. Euclidian Distance Mask (ED)

The influence of blanking is shown in Table 3, for several type of RFI. After correction, the signal has a level close to that corresponding to the natural microwave emission.

**Figure 7.**
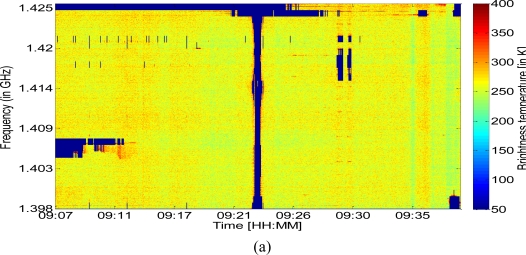

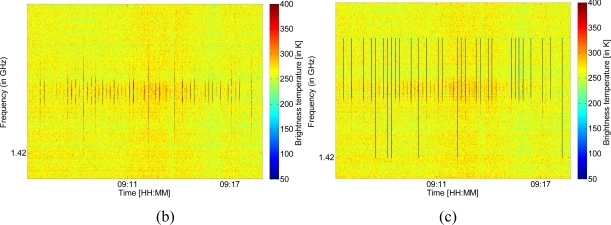
Brightness temperatures after correction using the Euclidian Filter, when the spectrum is divided into 32 sub-bands. The full set of data is shown in **(a)**, whereas an enlarged view of the pulsed RFI, before and after blanking, can be seen in **(b)** and **(c)** respectively.

Figure 7 illustrates the resulting time—frequency brightness temperature maps, before and after elimination of the corrupted sub-bands during the studied flight. The removed sub-bands are clearly seen, revealing the heterogeneity of the brightness temperature as a function of frequency. This result demonstrates the ability of the algorithm to eliminate corrupted sub-bands. For some other zones, such as the area around Auch, where a large part of the frequency band is corrupted by RFI, we did not keep the data.

### 5.3. Algorithm Comparisons

In this paragraph, we compare the two different RFI mitigation algorithms. Two factors are used in this analysis: the number of deleted data, and the number of mean Tb computed for only one small part of the transect, corresponding to a path length of approximately 40 km (close to the dimensions of the central field of view of a SMOS pixel). These values are presented in the following Table 4: the Euclidian algorithm was tested for a number of spectrum sub-bands ranging from 8 to 64.

In this table, it can be seen that mean Tb values determined for a ‘SMOS equivalent’ transect vary from 270.7 K to 261.4 K, depending on the applied mask. The most efficient mask was that based on the ED criterion, using 16 sub-bands. This mask allowed only 8.3% of the data to be deleted. In that case, a low percentage of data is deleted and a low mean Tb value is reached. Because of these low mean Tb value, we can suppose that this mask deletes most of RFI (which should increase mean Tb); in the meantime, low percentage means that, compared to other masks, less false alarms are present. The kurtosis mask led to a higher mean Tb value, with a larger amount of deleted data. This outcome can be interpreted as these algorithms also mask some uncorrupted parts of the spectrum.

**Figure 8.**
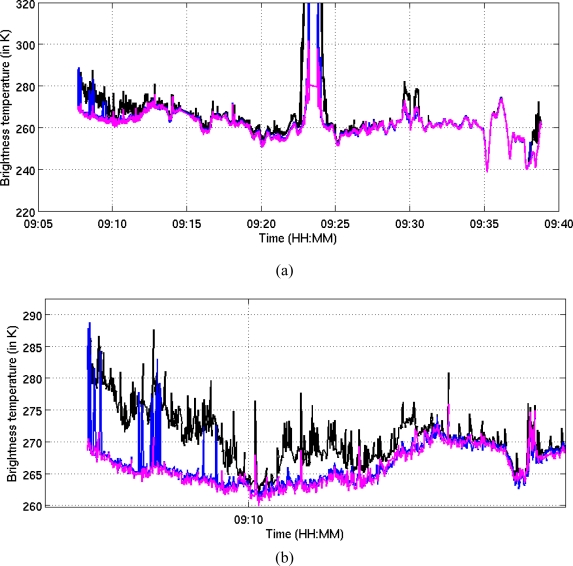
**(a)** Comparison of the different Tb values measured along the full length of the transect. **(b)** zoom on (a), near to the CEL site. The spectra are divided into 8 sub-bands in this figure. Black curve: no-blanking, blue: kurtosis, magenta: Euclidian distance.

Another way of comparing the algorithms is to compute the Tb values over the full L-band spectrum, before and after RFI masking. This comparison is made in Figure 8(a,b), which show the good accordance of these three algorithms. They all mitigate the RFI transmitted in the vicinity of the CEL site [zoom on Figure 8(b)], although the kurtosis algorithm did not efficiently mask all parts of this RFI. All the algorithms failed to eliminate the CW RFI detected near Auch, but succeeded in masking the RFI effects produced by switch-on of the on-board visible camera (around 9:28).

## 6. Discussion and Conclusions

A FFT spectrum analyzer has been developed, and used to complete the French L-band CAROLS radiometer receiver, with the objective to retrieve and remove RFI from the radiometric measurements. Our study was based on airborne CAROLS measurements acquired over southwest France. Spectral analysis of the data reveals two types of RFI, the first one—such as that observed near Auch—is broadband. The second one is produced by radar pulse emissions, and is narrowband. Following RFI correction, a new “clean” brightness temperature map, with uncorrupted sub-bands, is produced. The results from L-band observations clearly demonstrate the efficiency of the Euclidian distance algorithm, for RFI mitigation based on frequency domain blanking. Pulsed and continuous wave RFI are removed, and the brightness temperature can be correctly retrieved. This is an important result, because ocean salinity and surface soil moisture can thus be correctly estimated, even if the measurements are corrupted by RFI. We show that the other algorithm, based on kurtosis parameters, is also able to mitigate RFI on a spectral basis, with the drawback that some of the continuous waves RFI are not deleted. In the future, the Euclidian distance could be replaced by the Mahalanobis distance, calculated on the basis of polarimetric information related to subband and polarization diversity. This algorithm could improve the accuracy with which RFI and non-RFI samples are classified

## Figures and Tables

**Table 1.** Specifications of the CAROLS radiometer.

**Radiometer**	Total power
**Frequency**	1.400–1.427 GHz (−60 dB bandwidth)
**Frequency sampling**	139.4 MHz
**Stability**	0.1 K over 15 mn
**Sensitivity**	0.1 K for 1 s integration time
**Integration**	1 ms and 1.8 μs
**Calibration**	Internal load and noise diode
**Antenna**	Potter horn (no sidelobe) with 37.6° HPBW
**Temperature regulation (analog front end)**	45 °C ± 0.1 K

**Table 2.** Spectrum analyzer specifications.

**Frequency bandwidth**	1.3978–1.4268 GHz
**Number of integrations**	1,024
**Recording time**	72.01 ms
**FFT**	4K points
**Windowing**	Hamming
**Resolution of the FFT**	14.16 kHz

**Table 3.** Mean Tb values, before and after blanking, using the Euclidian distance, for each type of RFI.

	**Natural emission**	**Pulsed RFI**	**CW RFI in 3MHz BW**

**Mean Tb values**	267.2 K	268.2 K	279.4 K
**Mean Tb values after blanking**	267.2 K	267.5 K	270.0 K

**Table 4.** % of deleted data and mean Tb values, for two different masking algorithms, and division of the spectrum into 8, 16, 32 and 64 sub-bands.

	**Number of sub-bands**	**% of deleted data**	**Mean Tb value on a equivalent ‘SMOS pixel’**

Without Blanking kurtosis (β_4_)			270.7
	8	32.2	266.3

Euclidian Distance (ED)	8	13.0	265.8
	16	8.3	261.4
	32	5.8	261.6
	64	4.0	261.9
